# Ileal amino acid digestibility, and the endogenous enzyme activities in cannulated early growing pig fed soybean meal diets supplemented with β-mannanase

**DOI:** 10.5713/ab.260287

**Published:** 2026-07-27

**Authors:** So Dam Choi, Habeeb Tajudeen, Jun Young Mun, Abdolreza Hosseindoust, Priscilla Neves Silvestre, Sang Sik Lee, Jin-woo Jhoo, Min Ju Kim, Young In Kim, Jin Soo Kim

**Affiliations:** 1 Department of Animal Industry Convergence, Kangwon National University, Chuncheon, Korea; 2 Department of Applied Animal Science, College of Animal Life Science, Kangwon National University, Chuncheon, Korea; 3 School of Animal Life Convergence Science, Hankyong National University, Anseong, Korea; 4 Institute of Applied Humanimal Science, Hankyong National University, Anseong, Korea; 5 CTC Bio, Inc., Seoul, Korea

**Keywords:** Amino Acid Digestibility, Crude Protein, Ileum, Protease, Standardized Ileal Digestibility, Viscosity

## Abstract

**Objective:**

This study evaluated the effects of dietary β-mannanase supplementation on apparent and standardized ileal amino acid digestibility, endogenous digestive enzyme activity, and digesta viscosity in early growing pigs.

**Methods:**

Five 49-day-old Landrace×Yorkshire×Duroc intact male pigs (20.25±0.33 kg body weight) were surgically fitted with distal ileum T-cannula. Following a 14-day recovery and 4-day adaptation, ileal digesta was collected and stored at −20°C for further analyses. One pig was assigned a nitrogen-free diet; the remaining four pigs were assigned to two dietary treatments in a Latin square design: a control diet without β-mannanase (MS−) and a diet supplemented with 0.05% β-mannanase (MS+; 400 U/kg feed).

**Results:**

MS+ significantly increased dry matter (DM) and crude protein (CP) digestibility (p<0.05), with a tendency toward higher organic matter digestibility (p = 0.063). The apparent and standardized ileal digestibility (SID) of arginine, lysine, and threonine were significantly higher (p<0.05) in the MS+ group, with tendencies toward greater SID of isoleucine (p = 0.066), proline (p = 0.076), and tyrosine (p = 0.062). Activities of trypsin, protease, and amylase were greater (p<0.05) in MS+ pigs. Additionally, MS+ reduced jejunal digesta viscosity (p<0.05) and tended to reduce fecal viscosity (p = 0.074). Correlation analysis indicated that jejunal and fecal viscosities were negatively associated with DM and CP digestibility and the SID of tryptophan and valine. Conversely, trypsin and protease activities exhibited strong positive correlations with CP digestibility and the SID of valine, while amylase activity was positively tied to DM digestibility.

**Conclusion:**

Dietary supplementation with 0.05% β-mannanase (400 U/kg) in early growing pigs significantly improves the apparent ileal digestibility of DM and CP, as well as the SID of arginine, lysine, and threonine, by inducing a 25.0% reduction in jejunal viscosity that facilitates enzyme-substrate interactions and stimulates endogenous digestive enzyme secretion.

## INTRODUCTION

Soybean meal (SBM) is one of the most widely used protein sources in swine diets due to its high protein content and favorable amino acid profile. In addition to protein, SBM contains substantial amounts of carbohydrates in the form of non-starch polysaccharides (NSP) and oligosaccharides [1]. These NSP fractions include insoluble components such as cellulose, and soluble fractions composed primarily of pectic polymers and galactomannans [2,3]. In monogastric animals, NSP are generally regarded as anti-nutritional factors because endogenous digestive enzymes are unable to degrade these complex carbohydrates [4–6]. As a major soluble NSP component present in SBM, β-mannan has been shown to increase digesta viscosity and interfere with nutrient digestion and absorption [7]. Elevated viscosity reduces the efficiency of enzyme–substrate interactions and can alter digesta passage rate, ultimately impairing nutrient utilization [8].

To mitigate these limitations, the supplementation of exogenous enzymes in swine diets has been extensively investigated [9]. Among these, β-mannanase has received considerable attention for its ability to hydrolyze β-mannan into smaller oligosaccharides, thereby reducing the anti-nutritional effects of this polysaccharide [10]. Previous studies have demonstrated that β-mannanase supplementation can improve growth performance, nutrient digestibility, and metabolic responses in pigs fed SBM-based diets [11]. In addition to improving nutrient availability, exogenous enzymes may also influence digestive physiology by interacting with endogenous enzyme systems [12]. Enhanced degradation of NSP may increase the accessibility of nutrients in the digesta matrix, which in turn stimulates secretion and activity of endogenous digestive enzymes [13]. Despite a growing body of research on β-mannanase supplementation, information regarding its interaction with endogenous digestive enzymes and the associated changes in amino acid digestibility remains limited [14].

Furthermore, although reductions in digesta viscosity are frequently proposed as a primary mechanism underlying the beneficial effects of β-mannanase, the relationship among viscosity, endogenous enzyme activity, and nutrient digestibility has not been fully characterized, particularly in diets where SBM serves as the sole protein source [15]. Understanding these interactions is important for optimizing enzyme supplementation strategies and improving feed efficiency in swine production [16].

Therefore, the objective of the present study was to evaluate the effects of dietary β-mannanase supplementation on apparent and standardized ileal digestibility (SID) of amino acids, endogenous digestive enzyme activity, and digesta viscosity in cannulated pigs fed SBM-based diets in which SBM was the sole protein source.

## MATERIALS AND METHODS

### Animals, diets, and experimental design

The experiment was conducted at the Teaching and Research Farm of Kangwon National University, Chuncheon, Republic of Korea. Five 49-day-old Landrace×Yorkshire×Duroc (LYD) intact male pigs with an initial BW of 20.25±0.33 kg were used. Pigs were individually housed in pens (0.8×0.5 m) with concrete flooring and partially slated areas, and all pigs were fed their assigned diets individually. SBM was the sole source of dietary protein in all experimental diets, ensuring that the observed effects could be attributed exclusively to SBM-derived factors.

One pig was assigned a nitrogen-free diet throughout the entire experiment to determine basal endogenous ileal AA losses, which were used to calculate SID values. The remaining four pigs were randomly assigned to two dietary treatments in a 4×2 Latin square design with two periods: SBM-based control diet without β-mannanase (MS−) and SBM-based diet supplemented with 0.05% β-mannanase (MS+; equivalent to 400 U/kg feed). The β-mannanase product was donated by CTC Bio and is thermostable up to 90°C; the product contains 800,000 U/kg and was produced through fermentation with *Bacillus* spp. WL1 (patented in the United States and Republic of Korea). The 0.05% inclusion level, providing 400 U/kg feed, was selected based on the findings of Upadhaya et al. [4] and Jeon et al. [17], in which this dose improved amino acid and nutrient digestibility in growing pigs fed SBM-based diets. The rationale for using this dosage is supported by prior dose-response data indicating significant improvements in digestibility at 400 U/kg [11].

The experiment lasted 35 days in total. Each experimental period spanned 7 days, consisting of a 4-day adaptation phase followed by a 3-day ileal digesta collection. Diets were formulated to meet or exceed the nutritional requirements of 20–50 kg growing pigs according to NRC (Table 1) [18]. Pantothenic acid was not included in the vitamin premix and was supplied separately to meet the NRC requirement of growing pigs. Room temperature was maintained at 22°C under a 20 h light:4 h dark photoperiod. Feed was offered at a daily allowance of 2.5 times the estimated energy requirement for maintenance (2.5×197 kcal metabolizable energy/kg BW0.60) [18], divided into two equal meals at 09:00 and 17:00. Fresh drinking water was provided ad libitum throughout the experiment.

### Cannulation

All pigs underwent surgical cannulation of the distal ileum following intramuscular administration of a combination of tiletamine hydrochloride and zolazepam hydrochloride (0.2 mL/kg; Zoletil 50; Virbac Laboratories). Simple T-cannulas were implanted at the distal ileum using the procedure described by Jørgensen et al. [19]. Pigs were allowed 14 days of post-surgical recovery before the experiment commenced.

### Sample collection

The first four days of each experimental period served as a dietary adaptation phase. On day 4, chromic oxide (Cr_2_O_3_; 0.25%) was incorporated into the feed as an indigestible marker. Fecal samples were collected daily to confirm marker appearance. On day 7, a second 0.25% Cr_2_O_3_ marker was added to the morning meal, and quantitative ileal digesta collection continued until the second marker appeared, following the marker-to-marker methodology described by Kim et al. [20]. Ileal digesta samples were collected at 11:00 and 19:00, and all collected samples were immediately stored at −20°C until analysis. Additional ileal digesta samples were collected at the end of the experiment for enzyme activity assays.

### Nutrient digestibility calculations

The apparent ileal digestibility (AID) and SID of amino acids were calculated using the following equations [21]:


(1) $$\text{AID }(\%)=(1-[{\text{AA}}_{\text{digesta}}/{\text{AA}}_{\text{diet}}]\times [{\text{Cr}}_{\text{diet}}/{\text{Cr}}_{\text{digesta}}])\times 100$$

(2) $${\text{IAA}}_{\text{end}}=({\text{AA}}_{\text{digesta}})\times ({\text{Cr}}_{\text{diet}}/{\text{Cr}}_{\text{digesta}})$$

(3) $$\text{SID }(\%)=\text{AID}+({\text{IAA}}_{\text{end}}/{\text{AA}}_{\text{diet}})\times 100$$

where AID is expressed on a dry matter (DM) basis; AA_diet_ and AA_digesta_ are the AA concentrations of the diet and ileal digesta, respectively (g/kg DM); Cr_diet_ and Cr_digesta_ are the Cr_2_O_3_ concentrations of the diet and ileal output, respectively (g/kg DM); and IAA_end_ is the basal ileal endogenous AA loss (g/kg DM intake) determined using the nitrogen-free diet [21].

### Chemical analyses

The gross energy (GE) of diet and fecal samples was measured using a bomb calorimeter (Model 1241; Parr Instrument). Concentrations of DM (method 930.15), crude protein (CP; method 990.03), and ether extract (EE; method 2003.05) of all diet and excreta samples were determined in triplicate according to AOAC International [22]. Organic matter (OM) content was calculated indirectly by subtracting ash from DM. Chromic oxide was quantified using an automated spectrophotometer (Jasco V-650; Jasco) following the procedure of Jeon et al. [17].

The AA composition of feed and ileal digesta samples was determined by high-performance liquid chromatography (Waters 486; Waters) after acid hydrolysis in 6 N HCl at 110°C for 24 h [22]. Methionine and cysteine were determined separately following performic acid oxidation prior to hydrolysis [23]. Jejunal digesta viscosity was measured on the same day of collection following the procedure described by Sánchez-Uribe et al. [24]. Digesta were homogenized and transferred to 15 mL tubes in four replicate aliquots. Tubes were centrifuged at 1,500×g for 5 min at 4°C, and the resulting supernatant was subsequently centrifuged at 10,000×g for 10 min at 4°C. The second supernatant was held on ice and viscosity was measured immediately using a Brookfield Digital Viscometer (Model DVII, ver. 2.0; Brookfield Engineering Laboratories) calibrated to 25°C with 1.0 mL of supernatant per reading.

Digesta enzyme activities of trypsin, chymotrypsin, protease, amylase, and lipase were quantified from ileal digesta samples collected from all pigs using commercial assay kits: Trypsin (ab102531), Chymotrypsin (ab234051), Protease (ab111750), Amylase (ab102523), and Lipase (ab102524) (all from Abcam), following the manufacturer’s instructions.

### Statistical analysis

Data were analyzed using SAS ver. 9.4 (SAS Institute). The MIXED procedure was employed to evaluate dietary treatments, with the individual pig serving as the experimental unit. Dietary treatment was specified as a fixed effect, whereas replication, period nested within replication, and animal nested within replication were modeled as random effects. Pearson correlation analysis was performed using the SAS to evaluate the relationships between nutrient digestibility parameters and digestive enzyme activities. Statistical significance was declared at p<0.05, and differences with 0.05≤p<0.10 were considered as tendencies.

## RESULTS

### Nutrient digestibility

The digestibility of DM and CP was greater (p<0.05) in pigs fed the MS+ diet (Table 2). Additionally, OM digestibility tended to be greater in MS+ pigs (p = 0.063). No differences were observed in GE or EE digestibility between treatments.

### Apparent and standardized ileal amino acid digestibility

As shown in Table 3, the AID of arginine, lysine, and threonine was significantly greater (p<0.05) in pigs fed MS+. A tendency toward greater AID of isoleucine was also observed in MS+ pigs (p = 0.066). The AID of histidine, leucine, methionine, phenylalanine, tryptophan, and valine did not differ significantly between treatments. Among nonessential amino acids, tendencies toward greater AID were noted for proline (p = 0.076) and tyrosine (p = 0.062) in MS+ pigs, whereas alanine, aspartate, cysteine, glutamate, glycine, and serine did not differ between treatments.

Similarly, the SID of arginine, lysine, and threonine was significantly greater (p<0.05) in the MS+ group (Table 4), with tendencies for isoleucine (p = 0.066), proline (p = 0.076), and tyrosine (p = 0.062). The SID of alanine, aspartate, cysteine, glutamate, glycine, and serine showed no significant differences between treatments.

### Digestive enzyme activity

The effects of β-mannanase supplementation on enzyme activity in ileal digesta are presented in Figure 1. Activities of trypsin, protease, and amylase were greater (p<0.05) in the MS+ group than in MS− pigs. Activities of chymotrypsin and lipase did not differ significantly between treatments.

### Digesta and fecal viscosity

The effects of β-mannanase on digesta and fecal viscosity are shown in Figure 2. Jejunal digesta viscosity was significantly lower (p<0.05) in MS+ pigs than in MS− pigs. Fecal viscosity also showed a decreasing trend in MS+ pigs (p = 0.074).

### Correlation analysis

Correlation analysis indicated that jejunal digesta viscosity was negatively associated with DM and CP digestibility, alongside the SID of tryptophan and valine (Figure 3). Similarly, fecal viscosity was negatively correlated with DM and CP digestibility, as well as the SID of methionine, threonine, tryptophan, and valine. Conversely, trypsin activity exhibited strong positive correlations with DM and CP digestibility and the SID of arginine, isoleucine, leucine, lysine, threonine, and tryptophan. Furthermore, elevated protease activity was closely linked to higher CP digestibility and the SID of methionine, tryptophan, and valine, while increased amylase activity was strongly correlated with DM digestibility.

## DISCUSSION

The NSP present in SBM are recognized as anti-nutritional factors in monogastric animals because endogenous enzymes cannot hydrolyze these polysaccharides [24]. Elevated digesta viscosity resulting from soluble NSP physically limits the diffusion of digestive enzymes toward dietary substrates, thereby reducing overall nutrient digestibility [2,5]. The dietary inclusion of exogenous carbohydrase such as β-mannanase represents a well-established nutritional strategy to counter these effects in pigs [25].

In the present study, β-mannanase supplementation increased trypsin, protease, and amylase activities in ileal digesta [26]. Similar stimulation of endogenous enzyme secretion and activity following exogenous enzyme supplementation has been reported in growing pigs by Galli et al. [27] and Jang et al. [28]. Jang et al. [29] demonstrated that exogenous β-mannanase significantly reduced the incidence of diarrhea, further validating that the enzymatic degradation of soluble NSP plays a crucial role in stabilizing the gastrointestinal environment and improving overall digestive efficiency in young pigs. Enzymatic degradation of β-mannan disrupts the structural matrix of the ingesta, increasing nutrient accessibility and thereby stimulating a greater secretory response of pancreatic and intestinal enzymes [30].

The enhancement of endogenous enzyme activity observed in the present study aligns with previous findings indicating that elevated digestive enzyme levels are closely linked to improvements in nutrient and amino acid digestibility [31]. Protein digestion in pigs involves coordinated secretion and activation of proteolytic enzymes in the small intestine, where trypsin and related proteases hydrolyze dietary proteins into smaller peptides and free amino acids available for intestinal absorption [32,33]. Amylase activity, which contributes to carbohydrate hydrolysis, reduces the accumulation of undigested substrates in the digesta matrix, thereby reducing competition between carbohydrate and protein fractions for enzymatic processing [25,33]. The concurrent increases in trypsin, protease, and amylase activities observed in the MS+ group in this study provide a mechanistic basis for the improvements in the AID and SID of several amino acids, including arginine, lysine, and threonine [3,17]. The positive effects of β-mannanase supplementation on nutrient digestibility are also attributable to the reduction in digesta viscosity. Dietary supplementation with β-Mannanase hydrolyzes β-mannan into shorter oligomeric fragments, reducing the viscosity of intestinal contents [34]. Lower digesta viscosity enhances nutrient diffusion and facilitates greater contact between digestive enzymes and dietary substrates, which promotes more efficient digestion and absorption [35]. The present results are consistent with this mechanism: MS+ pigs showed both reduced jejunal digesta viscosity and improved AID and SID values for several amino acids compared with controls [36]. The reduction in digesta viscosity also improves the overall physicochemical environment of the gastrointestinal tract [26]. Highly viscous digesta restricts nutrient transport and limits uniform mixing of feed components with endogenous secretions, while lower viscosity promotes greater homogeneity of the luminal contents and more effective enzyme–substrate interactions [35,36]. These changes contribute collectively to the improvement in overall digestive efficiency observed in pigs fed diets high in SBM-derived NSP [2].

To further characterize the viscosity response to β-mannanase supplementation, digesta and fecal viscosity were measured. The present findings showed significantly lower jejunal digesta viscosity and a tendency toward reduced fecal viscosity in MS+ pigs, consistent with the ability of β-mannanase to degrade β-mannan into smaller polymer units of lower viscosity [15]. As molecular chain length decreases, the capacity of polysaccharide molecules to entangle with adjacent molecules and exert friction within the fluid also decreases, resulting in lower viscosity [37]. In a dose-response study using nursery pigs, Jang et al. [36] reported that broken-line regression analysis indicated that jejunal digesta viscosity was significantly reduced at a β-mannanase supplementation level of 360 U/kg feed. The 400 U/kg dose used in the present study exceeds this threshold and is consistent with the significant viscosity reduction observed here. The degree of polymerization at which mannan oligosaccharides no longer exert viscosity-elevating effects warrants further investigation.

As noted, elevated digesta viscosity acts as a physical barrier, impeding the diffusion and adsorption of digestive enzymes onto their respective dietary substrates [35]. The present correlation data strongly support this primary mechanism, demonstrating that an increase in digesta viscosity directly suppresses the functional activities of trypsin, protease, and amylase within the lumen. Consequently, this enzymatic inhibition leads to a targeted reduction in the digestibility of arginine, lysine, and threonine. The selective improvements in arginine and lysine can be biochemically justified by the substrate specificity of trypsin [38], which exclusively cleaves peptide bonds at the carboxyl side of these two basic amino acids. Furthermore, the strong correlation between chymotrypsin activity and arginine, lysine digestibility may be explained by the reduced secretion of endogenous mucin proteins [39], as lower viscosity decreases physical friction against the intestinal mucosa. Taken together, these multivariate outcomes successfully establish that dietary β-mannanase promotes amino acid utilization through a coordinated triad mechanism: lowering luminal viscosity, stimulating key endogenous proteases, and subsequently maximizing substrate-specific amino acid liberation.

## CONCLUSION

Supplementing a SBM-based diet with 0.05% β-mannanase (400 U/kg) in early growing pigs significantly improves the AID of DM and crude protein, as well as the SID of arginine, lysine, and threonine. This nutritional benefit is directly driven by a 25.0% reduction in jejunal digesta viscosity, which biologically correlates with enhanced endogenous trypsin, protease, and amylase activities. These outcomes establish a causal mechanism where reduced digesta viscosity eliminates physical barriers in the lumen, thereby optimizing enzyme-substrate interactions and maximizing specific amino acid liberation in the small intestine.

## Figures and Tables

**Figure 1:**
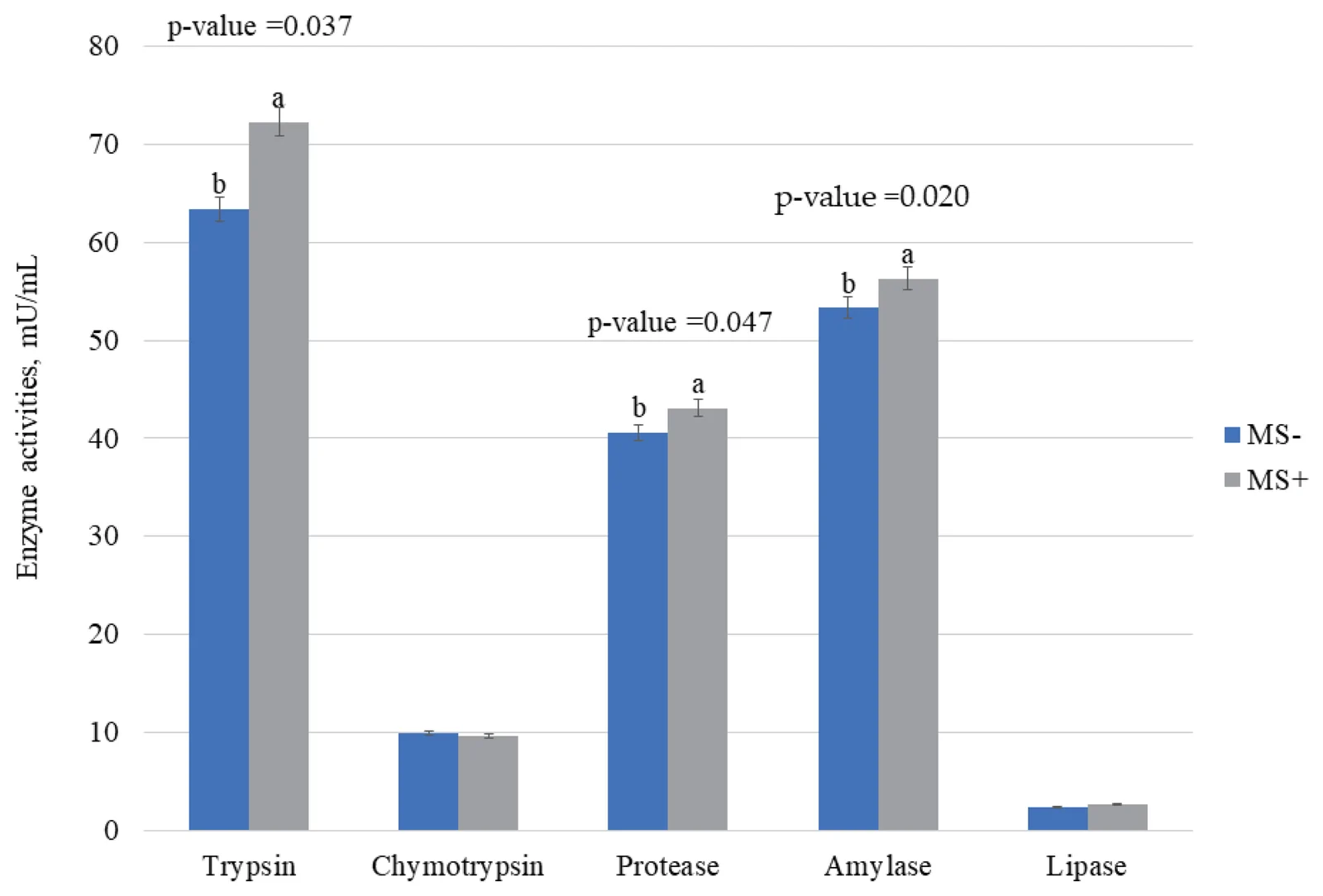
The effects of β-mannanase supplementation on the enzyme activity of cannulated early growing pigs. ^a,b^ Means with different lowercase letters differ significantly (p<0.05). MS−, soybean meal without mannanase; MS+, soybean meal with 0.05% β-mannanase.

**Figure 2:**
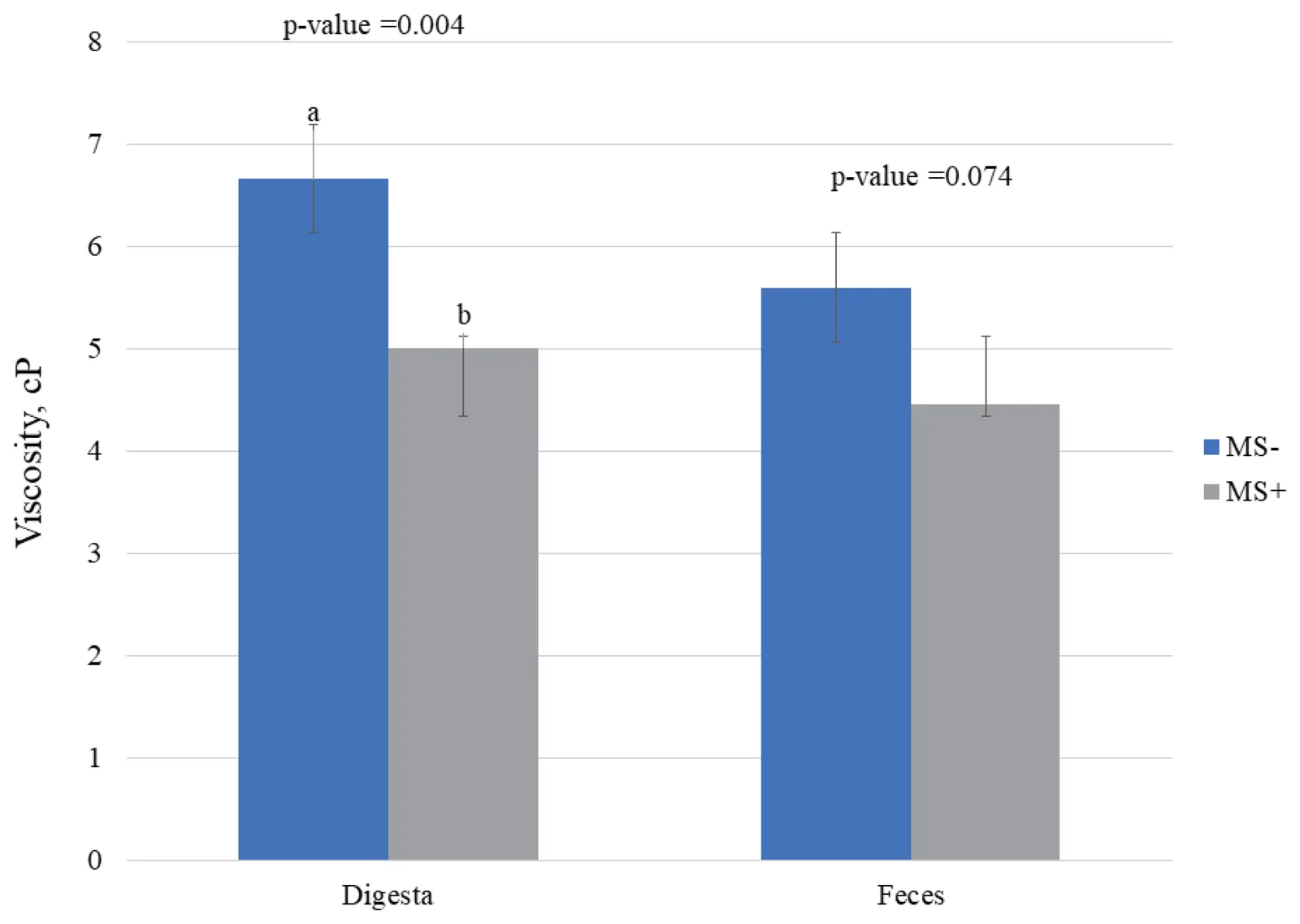
The effects of β-mannanase supplementation on digesta and fecal viscosity in cannulated early growing pigs. ^a,b^ Means with different lowercase letters differ significantly (p<0.05). MS−, soybean meal without mannanase; MS+, soybean meal with 0.05% β-mannanase.

**Figure 3:**
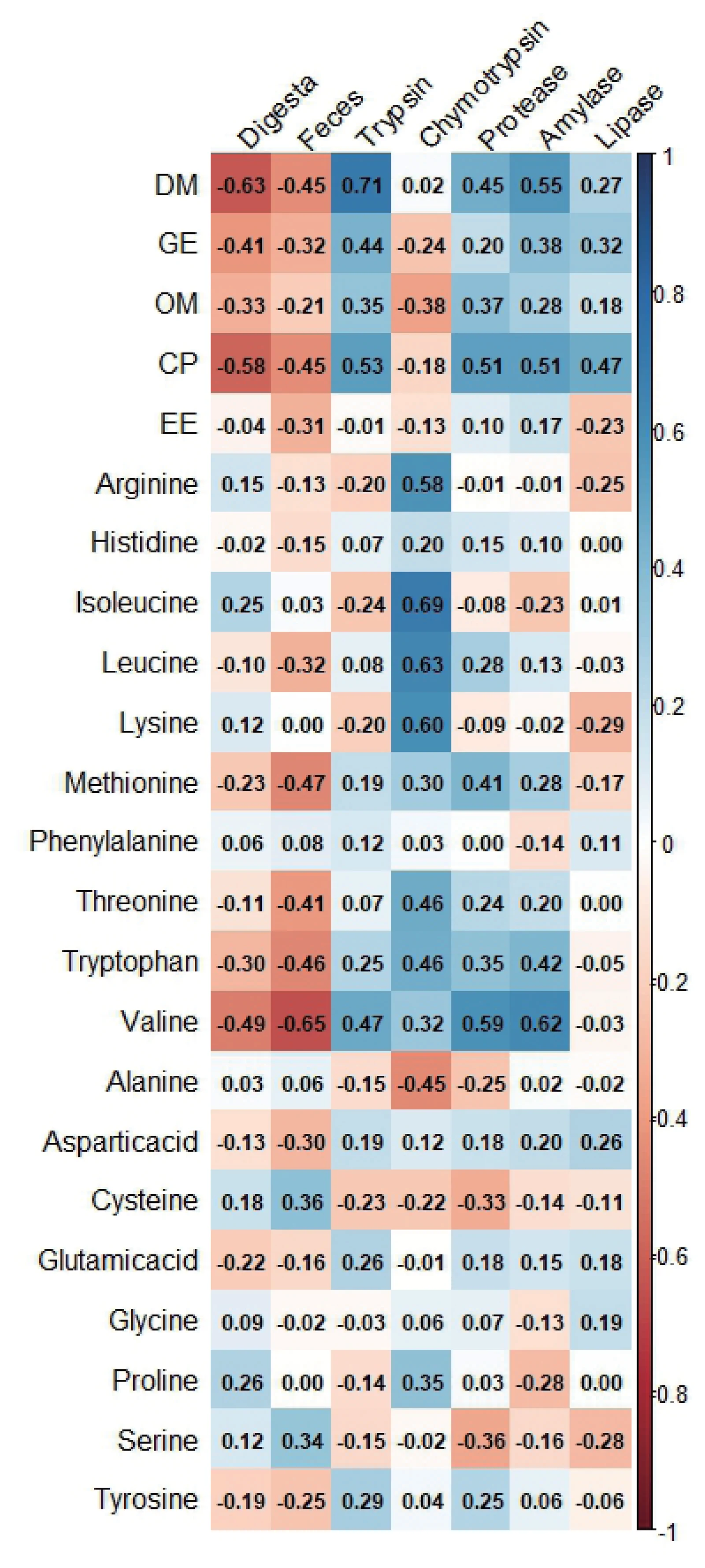
Correlation heatmap matrix between digestive parameters. The abscissa represents endogenous digestive enzyme activities and digesta viscosities, while the ordinate represents the apparent ileal digestibility of proximate nutrients and the standardized ileal digestibility of amino acids. Color intensity and the displayed numbers represent the Pearson correlation coefficients (r). Blue indicates a positive correlation, while red indicates a negative correlation. DM, dry matter; GE, gross energy; OM, organic matter; CP, crude protein; EE, ether extract.

**Table 1:** Composition and calculated contents of experimental diets (% as-fed basis)

Ingredients	N-free	Soybean meal
Corn starch	56.30	55.84
Glucose	20.00	-
Soybean meal	-	41.58
Sugar	20.00	0.00
Limestone	1.21	1.43
Monocalcium phosphate	1.34	-
Salt	0.30	0.30
Vitamin premix^1)^	0.30	0.30
Mineral premix^2)^	0.30	0.30
Chromium oxide	0.25	0.25
Calculated composition	-	-
ME (kcal/kg)	3,504	3,295
Crude protein	-	19.50
Calcium	0.70	0.70
Available phosphorus	0.33	0.33
Lysine	-	1.20
Methionine+cysteine	-	0.56

1) Provided per kg of diet: 16,000 IU vitamin A (palmitate), 2.00 mg vitamin B_1_ (thiamin), 5.00 mg vitamin B_2_ (riboflavin), 2.00 mg vitamin B_6_ (pyridoxine), 0.03 mg vitamin B_12_ (cyanocobalamin), 25.00 mg niacin, 0.40 mg folic acid, 0.05 mg biotin, 5.00 mg ethoxyquin, 2,000 IU vitamin D_3_ (cholecalciferol), 75.00 mg vitamin E (dl-α-tocopheryl acetate), 2.00 mg vitamin K_3_ (menadione).

2) Provided per kg of diet: 100 mg Fe, 6 mg Cu, 4 mg Mn, 0.3 mg Se, 0.14 mg I, 0.25 mg Co.

ME, metabolizable energy.

**Table 2:** Effects of β-mannanase supplementation on the nutrient digestibility of cannulated early growing pigs (%)

Item	MS−	MS+	SEM	p-value
Dry matter	79.73^b^	81.93^a^	0.71	0.021
Gross energy	78.68	79.78	1.20	0.177
Organic matter	79.95	81.01	0.80	0.063
Crude protein	80.01^b^	81.62^a^	1.01	0.017
Ether extract	68.27	68.18	0.98	0.891

a,b Means bearing different superscript letters differ significantly.

MS−, soybean meal without β-mannanase; MS+, soybean meal with 0.05% β-mannanase; SEM, standard error of the means.

**Table 3:** Effects of β-mannanase supplementation on apparent ileal amino acid digestibility in cannulated early growing pigs (%)

Amino acids	MS−	MS+	SEM	p-value
Essential				
Arginine	88.72^b^	91.52^a^	1.57	0.018
Histidine	86.65	87.78	2.71	0.522
Isoleucine	80.51	83.27	2.12	0.066
Leucine	82.40	83.60	1.65	0.276
Lysine	84.34^b^	86.60^a^	1.00	0.013
Methionine	80.75	83.01	1.72	0.363
Phenylalanine	82.23	81.18	1.65	0.338
Threonine	77.30^b^	81.10^a^	1.98	0.013
Tryptophan	80.38	80.08	1.82	0.800
Valine	76.68	74.80	2.34	0.233
Non-essential				
Alanine	82.26	84.35	4.27	0.456
Aspartate	78.06	73.97	8.24	0.450
Cysteine	80.60	81.99	3.04	0.486
Glutamate	85.24	82.97	2.05	0.110
Glycine	62.67	65.97	9.59	0.587
Proline	46.67	54.36	6.16	0.076
Serine	80.27	81.82	2.89	0.415
Tyrosine	81.77	77.79	2.99	0.062

a,b Means bearing different superscript letters differ significantly.

MS−, soybean meal without β-mannanase; MS+, soybean meal with 0.05% β-mannanase; SEM, standard error of the means.

**Table 4:** Effects of β-mannanase supplementation on standardized ileal amino acid digestibility in cannulated early growing pigs (%)

Amino acids	MS−	MS+	SEM	p-value
Essential				
Arginine	92.46^b^	95.25^a^	1.57	0.018
Histidine	89.24	90.38	2.71	0.522
Isoleucine	83.45	86.21	2.12	0.066
Leucine	85.53	86.74	1.65	0.276
Lysine	86.80^b^	89.05^a^	1.00	0.013
Methionine	87.08	89.35	1.72	0.363
Phenylalanine	85.22	84.17	1.65	0.338
Threonine	85.01^b^	88.81^a^	1.98	0.013
Tryptophan	88.44	88.14	1.82	0.800
Valine	78.06	76.18	2.34	0.233
Non-essential				
Alanine	89.06	91.47	4.27	0.456
Aspartate	113.75	109.65	8.24	0.450
Cysteine	89.00	90.39	3.04	0.486
Glutamate	88.79	86.52	2.05	0.110
Glycine	91.33	94.73	9.59	0.587
Proline	81.95	89.65	6.16	0.076
Serine	91.49	93.04	2.89	0.415
Tyrosine	90.85	86.88	2.99	0.062

a,b Means bearing different superscript letters differ significantly.

MS−, soybean meal without β-mannanase; MS+, soybean meal with 0.05% β-mannanase; SEM, standard error of the means.

## Data Availability

Upon reasonable request, the datasets of this study can be available from the corresponding author.
